# Análisis de la situación, evaluación y propuestas de mejora del Programa de Actividades Preventivas y de Promoción de la Salud (PAPPS)^☆^

**DOI:** 10.1016/j.aprim.2020.09.002

**Published:** 2020-12-30

**Authors:** Pilar Martín-Carrillo Domínguez, María Martín-Rabadán Muro, Jesús González-Lama, Esperanza Romero-Rodríguez, Luis Ángel Pérula de Torres, Francisco Camarelles Guillén

**Affiliations:** a Grupo de evaluación y mejora del PAPPS (semFYC); b Instituto Maimónides de Investigación Biomédica de Córdoba (IMIBIC), Hospital Universitario Reina Sofía, Universidad de Córdoba, Córdoba, España; c PAPPS y Grupo de educación para la salud del PAPPS (semFYC)

**Keywords:** Atención Primaria, Prevención, Promoción de salud, Programa de evaluación, Primary care, Prevention, Health Promotion, Evaluation program

## Abstract

**Objetivo:**

Analizar la situación, evaluación y propuestas de mejora del Programa de Actividades Preventivas y de Promoción de la Salud (PAPPS).

**Material y métodos:**

Se realizó un estudio cualitativo de análisis de la situación para una evaluación del PAPPS en 2 fases: 1) generación de ideas y recogida de información a través de una matriz DAFO, a través de 2 tipos de criterios, internos (fortalezas y debilidades), y externos (amenazas y oportunidades); 2) priorización de las propuestas de mejora recogidas. Selección de participantes: se identificaron informadores-clave teniendo en cuenta su relación y conocimiento del programa PAPPS. Se incluyeron a todos los integrantes de los grupos de expertos del PAPPS y miembros con participación en el pasado, así como a los componentes del organismo de coordinación, incluidos los responsables autonómicos del PAPPS. Se enviaron dos invitaciones a participar en el estudio: la primera desde el 29 diciembre de 2017 al 11 febrero de 2018, y la segunda entre el 10 y el 23 de enero de 2019. La información se obtuvo a partir de un cuestionario concebido para ser autocumplimentado.

**Resultados y conclusiones:**

Respondieron el cuestionario un total de 73 sujetos. El 35% de los participantes eran miembros de los grupos de trabajo del PAPPS, siendo el grupo más numeroso, seguido de médicos de familia de otros ámbitos, con el 20,5%. El orden de priorización de las propuestas de mejora fue el siguiente: 1) unificar recomendaciones con otros grupos de trabajo de semFYC; 2) elaborar listados con «Recomendaciones No hacer» desde el punto de vista de la prevención; 3) incorporar el PAPPS en la agenda política; 4) mayor coordinación e interacción entre grupos con competencias comunes; 5) docencia en pregrado y en unidades docentes, darse a conocer también a los estudiantes de Medicina; 6) revisión, actualización y difusión a cualquier profesional de Atención Primaria.

## Abstract (en)

**Objective:**

To analyze the situation, evaluation and proposals for improvement of the Preventive Activities and Health Promotion Program (PAPPS).

**Material and methods:**

A qualitative study of situation analysis was carried out for the evaluation of the PAPPS in 2 phases: 1) Generation of ideas and collection of information through a DAFO matrix, using 2 types of criteria: internal (strengths and weaknesses), and external (threats and opportunities); 2) Prioritization of the improvement proposals collected. Selection of participants: Key informants were identified taking into account their relationship and knowledge of the PAPPS program. All members of the PAPPS, expert groups and members with past participation were included, as well as the coordinators, including the autonomous leaders of the PAPPS. Two invitations to participate in the study were sent: the first from December 29, 2017 to February 11, 2018, and the second from January 10 to 23, 2019. The information was obtained from a questionnaire designed to be self-completed.

**Results and conclusions:**

A total of 73 subjects answered the questionnaire. 35% of the participants were members of the PAPPS working groups, followed by family doctors from other areas, with 20.5%. The order of prioritization of the improvement proposals was as follows: 1) Unify recommendations with other semFYC working groups; 2) Prepare lists with “Recommendations not to do” from the point of view of prevention; 3) Incorporate PAPPS in the political agenda; 4) Greater coordination and interaction between groups with common competences; 5) Teaching in undergraduate and teaching units; 6) Review, update and dissemination of the program in Primary Care.

## Introducción

El Programa de Actividades Preventivas y de Promoción de la Salud (PAPPS) es un proyecto de la Sociedad Española de Medicina de Familia y Comunitaria (semFYC, 1982), que se creó en 1988 con el objetivo de promover la salud y las prácticas preventivas en Atención Primaria (AP) en nuestro país^1^. Los impulsores del PAPPS se inspiraron en los programas de prevención Canadian Task Force on Preventive Health Care^2^ (1978) y United States Preventive Services Task Force^3^ (1984).

Desde su inicio, el PAPPS ha promovido el fomento de la calidad asistencial en los centros de AP a través de la integración de un programa de actividades preventivas y de promoción de la salud de la población, la identificación de las dificultades que genera su implantación y las necesidades organizativas para ponerlo en práctica, proporcionando recomendaciones preventivas periódicas basadas en la evidencia científica e informando de los recursos disponibles y de los resultados de las evaluaciones de las actividades preventivas en AP, y fomentando la formación y la investigación sobre la prevención en AP^1^.

En 2005, se desarrolló el primer análisis sobre el impacto y las dificultades que el PAPPS había experimentado desde su instauración^4^. El estudio incluyó un análisis cualitativo (a través de una matriz DAFO), y cuantitativo, para conocer las opiniones de los profesionales sanitarios sobre el programa preventivo. Los resultados indicaron que el PAPPS había contribuido de manera sustancial a mejorar la calidad asistencial y el desarrollo de la AP en España, si bien se consideró necesario promover la dinamización del programa, con el objeto de incrementar la implicación de los profesionales en las recomendaciones preventivas.

En el citado análisis, se consensuaron 18 recomendaciones encaminadas a promover el desarrollo del PAPPS en el futuro, entre ellas cabe destacar su integración en el catálogo de prestaciones de los servicios de salud de las Comunidades Autónomas (carteras de servicios), fomentar en el ciudadano una mayor responsabilidad sobre el cuidado de su propia salud (*patient empowerment*), colaborar con otras sociedades científicas para generar y difundir recomendaciones consensuadas, o poner en marcha investigaciones con diseños más potentes para evaluar los resultados en salud de las distintas recomendaciones (ej. estudios multicéntricos de coste-efectividad), entre otras. Entre los factores que podían influir desfavorablemente en una mayor implantación de las recomendaciones preventivas se resaltaba la elevada presión asistencial o la escasez de tiempo disponible para llevar a cabo de manera adecuada las recomendaciones postuladas y la excesiva burocratización de las consultas de AP.

El Ciclo PDCA (o círculo de Deming)^5^ es la metodología más usada para implantar un sistema de mejora continua, cuyo principal objetivo es la autoevaluación, destacando los puntos fuertes que hay que tratar de mantener y las áreas de mejora en las que se deberá actuar. Tras casi 15 años del primer análisis evaluativo del programa, en el 2019 se planteó la realización de un nuevo estudio cualitativo con el objetivo de disponer de información que ayudara a facilitar la elaboración de un plan estratégico que permitiese definir, concretar y evaluar mejor las acciones a realizar para reactivar e impulsar la difusión y adopción de las medidas propuestas por los diferentes grupos de trabajo del PAPPS.

## Metodología

Se realizó un estudio cualitativo de análisis de la situación para una evaluación del PAPPS en 2 fases:

1) Generación de ideas y recogida de información a través de una matriz DAFO. Esta es una técnica que se aplica en los estudios evaluativos sobre dinámicas sociales participativas^6^, y resulta útil cuando se pretende impulsar transformaciones estructurales y dinamizar el cambio o elaborar proyectos de acción, como es el caso del PAPPS. Metodológicamente, la técnica DAFO se concreta en preguntas que corresponden a 2 tipos de criterios: internos (fortalezas y debilidades), y externos (amenazas y oportunidades).
2) Priorización de las propuestas de mejora recogidas.

### Selección de participantes

Se identificaron informadores-clave teniendo en cuenta su relación y conocimiento del programa PAPPS. Se incluyeron a todos los integrantes de los grupos de expertos del PAPPS y miembros con participación en el pasado, así como a los componentes del organismo de coordinación, incluidos los responsables autonómicos del PAPPS. También se invitó a colaborar a los profesionales pertenecientes a la junta directiva de la semFYC y a los integrantes de otros programas de la sociedad científica relacionados con actividades preventivas o promoción de la salud, como los del Grupo de Comunicación y Salud y del Programa de Actividades Comunitarias en Atención Primaria (PACAP). Así mismo se incluyeron responsables en temas de prevención/promoción de la salud de las distintas Comunidades Autónomas del estado español.

1. ª Fase. Aplicación de la matriz DAFO.

La invitación se realizó por correo electrónico a los candidatos elegidos. En el mensaje se explicaba el objetivo del estudio, animando a la participación. En el texto del mensaje se adjuntaba el enlace al cuestionario a cumplimentar. En la encuesta se incluían preguntas sobre cambios en el programa y acciones de mejora. El cuestionario se elaboró con la herramienta *Google Forms* en Drive (dicho formulario se puede consultar en el siguiente enlace: https://docs.google.com/forms/d/e/1FAIpQLSc3C3P_oO2Y9VZu_djMLf_JO2xUW9wqyh75GXDIEp1Fc9RJEA/viewform).

Mediante un enfoque de análisis cualitativo y estadística descriptiva, se realizó una síntesis de la información reagrupando las ideas y opiniones generadas en varias categorías.

2. ª Fase. Priorización de actividades sobre las propuestas de mejora recogidas.

Se realizó una presentación de los resultados obtenidos en las Jornadas del PAPPS, organizadas en el Ministerio de Sanidad y Consumo, en febrero de 2019, y a las que asistieron la mayor parte de los participantes de este estudio.

Tras la presentación de las propuestas recogidas en la fase anterior y en el seno de esta reunión se realizó una priorización de las mismas. Para ello se utilizó el programa Socrative^7^ que permite contestación simultánea de los participantes mediante sus teléfonos móviles. Se solicitó que puntuaran mediante una escala Likert con un rango de 1 a 3 las actividades o propuestas que considerasen como más prioritarias, asignándoles una mayor puntuación a las más importantes a implementar.

## Resultados

### Resultados obtenidos con el DAFO

**Figura 1:**
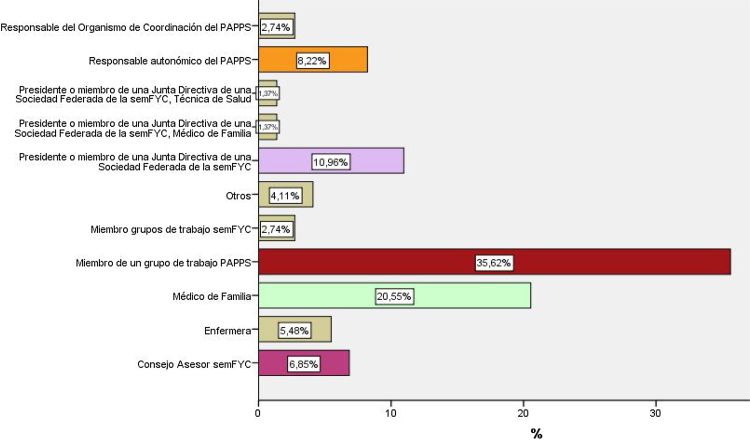
Participantes en la matriz DAFO según su actividad en relación al programa PAPPS.

**Figura 2:**
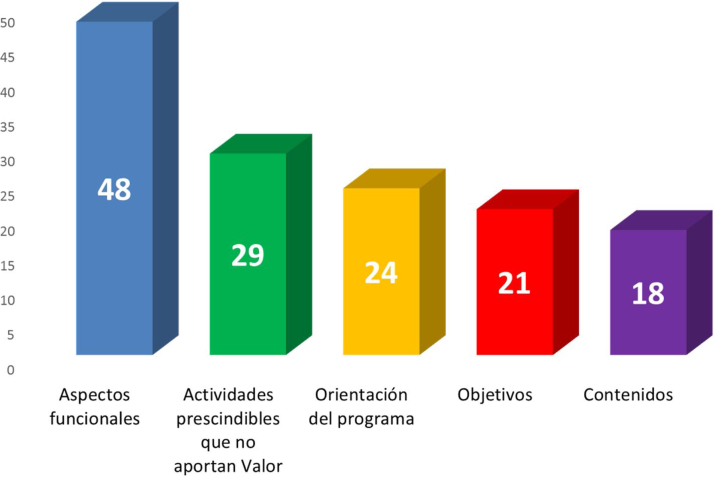
Categorías de agrupación de los cambios propuestos en el programa PAPPS (n).

Se realizaron dos envíos de correos electrónicos invitando a la participación; el primero entre el 29 diciembre de 2017 al 11 febrero de 2018, y el segundo entre el 10 y el 23 de enero de 2019. Respondieron a las preguntas del cuestionario para la elaboración de la matriz DAFO un total de 73 sujetos. En el primer envío se recibieron 41 cuestionarios, y en el segundo otras 32 respuestas. El 35% de los participantes eran miembros de los grupos de trabajo del PAPPS, siendo el grupo más numeroso, seguido de médicos de familia de otros ámbitos, con el 20,5% (fig. 1). Las respuestas, subdivididas en debilidades, amenazas, fortalezas y oportunidades obtenidas tras la síntesis y categorización de la información se exponen en la tabla 1. Los cambios propuestos se recogen en la figura 2.

**Tabla 1:** Respuestas obtenidas mediante la matriz DAFO (matriz de factores)

Análisis interno	Análisis externo
**Fortalezas**	**Oportunidades**
*1. Trayectoria del programa* Experiencia, larga y sólida trayectoria, 30 años de andadura Popularidad, implantación en las CC. AA. Ser un referente nacional e internacional Credibilidad, reconocimiento, reputación, liderazgo, prestigio Acceso abierto a la publicación bienal de actualización Llegar a un amplio colectivo Estar presente en otros grupos científicos a nivel internacional (WONCA, EUROPREV, Confederación Iberoamericana de Medicina Familiar) *2. Profesionales* Profesionalidad, responsabilidad, experiencia y compromiso de sus miembros Mucha gente colaborando Independencia, sin conflictos de intereses, transparencia Entusiasmo, tiempo dedicado *3. Del programa* Actualización, utilidad y valor para el Médico de Familia Rigor científico y metodológico, calidad científica, fiabilidad, recomendaciones basadas en pruebas, objetividad, evidencias científicas que la sustentan Su carácter sintético lo hace fácil de consultar Vehículo formativo Posibilidad de evaluación e investigación Las revisiones, recordatorio constante sobre la relevancia profesional de la Promoción de la Salud y la Educación para la Salud (sección magnífica del blog con comentarios y reflexiones) Su histórico Independencia, imparcialidad, no influencia de la industria Grupos de trabajo consolidados, con formación específica y capacidad de trabajo «expertos» en temas concretos Coordinación Necesita poco presupuesto para implementarse	El PAPPS es un programa de gran prestigio reconocido por las instituciones sanitarias del estado especialmente el Ministerio de Sanidad Consumo y Bienestar Social. Es un programa referente nacional en prevención y promoción de la salud Muchas de las recomendaciones de los diversos grupos del PAPPS se han oficializado en el ámbito del SNS Buena parte de las recomendaciones han sido incorporadas a las carteras de servicios de los distintas Servicios de salud de las Comunidades autónomas El programa mantiene relaciones internacionales con otros programas preventivos de países de nuestro entorno
**Debilidades**	**Amenazas**
*Conocimiento, influencia, visibilidad* Escasa difusión de sus recomendaciones Escasa visibilidad e influencia en: Médicos de familia, residentes: muchos desconocen el PAPPS Enfermeras En otras Sociedades científicas, incluso en semFYC y en la población Poca utilización de redes sociales en el plan de comunicación Página web antigua, poco amigable y poco atractiva, que debería estar dirigida también a la población Presentación obsoleta en revista Atención Primaria, no ágil para búsqueda de dudas No ha conseguido introducir la cultura de la prevención en los centros de salud *Gestión interna* Falta de innovación, estancado, necesita renovar su proyecto inicial Poco recambio generacional, necesita incorporación de gente joven (regeneración) Funcionamiento dispar de los responsables autonómicos (dar mayor protagonismo) No existe «plan estratégico», ¿Sabemos a dónde queremos ir? ¿Cuáles son nuestros objetivos a corto, medio y largo plazo? Falta comunicación y coordinación interna (pocas reuniones y las actas se retrasan dificultando su dinamismo) Falta sentido de pertenencia y cohesión de los miembros, con escaso trabajo en equipo y en red, con poco uso de nuevas tecnologías para comunicarse Falta de liderazgo y dinamización Basado en el voluntarismo y las iniciativas personales. Visibilidad y reconocimiento menguantes como grupo. *Relaciones con otros grupos, programas, profesiones y autoridades* Falta de coordinación con otros grupos de trabajo de semFYC (que deberían incluir miembros del PAPPS y así impedir recomendaciones discrepantes) Escasa coordinación con enfermeras, parte fundamental Falta de más vínculos con el PACAP y el establecimiento de líneas conjuntas de trabajo en Promoción de la salud Poca coordinación con los Servicios de Salud y el Ministerio de Sanidad *4. Recomendaciones PAPPS* La mayoría están incluidas en Cartera de Servicios del Sistema Nacional de Salud Pérdida de las evaluaciones periódicas. Desconocemos en qué fase estamos, y no nos damos a conocer a otros estamentos Escasa implementación y falta de información actualizada La diversidad de grupos y de recomendaciones, derivadas por la amplitud del ámbito que abarca Falta de una metodología estandarizada para aplicar las recomendaciones Contenido de las recomendaciones: teóricas, muy extensas en algunos temas, con actividades poco claras, no concisas, y escasamente realizables Conformarse con realizar la actualización bienal, sin más conexión entre sus miembros ni más objetivos en relación con la investigación o la formación *5. Otros* Contenidos poco conocidos, obsoletos, poco prácticos. La competencia con otros programas o información más «inminente», más atractiva en el formato de presentación No abrirse a otros colectivos No incidir en aspectos comunitarios y relativos a los pacientes Un grupo de trabajo muy cerrado a nuevas incorporaciones y con un nivel de exigencia alto que frena la entrada de jóvenes al mismo Desmotivación de sus integrantes y dirigentes Cierta endogamia y cansancio Necesidad de incorporar nuevos miembros jóvenes y activos, más abiertos a propuestas innovadoras *6. Apoyo y financiación* Falta de apoyo y recursos, tanto de la Junta directiva de semFYC, como de las Sociedades federadas Pérdida de autonomía por excesivo control desde la Junta directiva de semFYC Falta de una proyección estratégica de la «marca» PAPPS *7. Investigación* Falta de una línea clara de investigación, mantenida en el tiempo	*1.- Relaciones y apoyos* Falta de financiación externa Falta de apoyo de las administraciones, gran variabilidad entre CC. AA. Poca coordinación con los Servicios de Salud y el Ministerio de Sanidad Desaparecer en la Cartera de Servicios de las CC. AA. y perder la autoría de las recomendaciones Farmaindustria controla gran parte de la formación en Atención Primaria (AP) y no le interesa tanto la prevención Otras sociedades científicas de AP que «compiten» por los residentes de la especialidad Interés de industria farmacéutica por medicalizar nuestras vidas y obviar la prevención *2. Programa* La escasa coordinación en las campañas de prevención con AP producen una pérdida en la orientación individual de la prevención y desaparece el consejo médico personalizado Múltiples distorsiones y exageraciones en la prevención secundaria (cribados de próstata, mama, etc.) Corrientes de cuestionamiento científico y social de la importancia de la prevención Recomendaciones generadas por gestores y no por profesionales asistenciales Diversidad de historias clínicas informatizadas Excesos o tópicos infundados y obsoletos en la cultura de la prevención («Más vale prevenir que curar», «prevenir siempre es bueno») Los intereses comerciales y sociales: Potentes conflictos de intereses de colegas (supuestos «expertos» individuales) Algunas Sociedades científicas cuestionan las recomendaciones del PAPPS La influencia de la industria en las recomendaciones del PAPPS controvertidas y contradictorias Desarrollo de recomendaciones por otras sociedades científicas y otros «especialistas». La globalización que supone Google y otros servidores sanitarios Menor peso de la semFYC en el contexto médico actual *3. Profesional y gestión clínica* Pérdida de su rol de referente en España y otros países, y ser superado o incluso sustituido por otros programas Pérdida de credibilidad y confianza por parte de los profesionales de atención primaria Las generaciones más jóvenes conocen poco su historia y lo que ha representado el PAPPS. Riesgo de desaparición o de papel marginal La clínica se impone a la prevención. Aumento de la carga asistencial es un obstáculo para su aplicación real en las consultas Convertirse en rutina «académica», respetada pero no aplicada

### Priorización de actividades sobre las propuestas de mejora recogidas

En la tabla 2 se muestran las acciones de mejora propuestas para el programa PAPPS. Se obtuvieron 50 propuestas de acciones de mejora que se agruparon en las siguientes categorías (fig. 1):

1. Aspectos funcionales del programa.
2. Orientación del programa.
3. Objetivos del programa.
4. Cambios en las actividades del programa.
5. Otros contenidos.

**Tabla 2:** Acciones de mejora propuestas para el PAPPS

**Formación y docencia**
Formación en metodologías evaluativas a los socios de semFYC interesados Cursos formativos sobre sistematización de las revisiones para todos los grupos Elaborar listados con «Recomendaciones No hacer» desde el punto de vista de la prevención Nuevos cursos formativos de actualización. Cursos para ligarlo con la educación poblacional (mejoraría el uso de la AP y gestión de consulta) Materiales divulgativos para profesionales y población APP y formación online Talleres sobre actividades preventivas Cursos online de PAPPS para dar a conocer a residentes y jóvenes Médicos de Familia Docencia en pregrado y en unidades docentes. Darse a conocer también a los estudiantes de Medicina. Lanzar «píldoras periódicas» con recomendaciones concretas Mesa en el Congreso de la semFYC Blog de Salud y Prevención PAPPS Vídeos educativos a profesionales y pacientes
**Coordinación**
Mayor coordinación e interacción entre grupos con competencias comunes Reunión anual de todos los grupos conjuntamente Reafirmar más los grupos de trabajo Coordinación con otras sociedades científicas para conseguir amplios consensos sobre temas controvertidos Promover la participación de asociaciones de pacientes Compartir el PAPPS con sociedades de enfermería Establecer relaciones más estrechas con las organizaciones que tengan objetivos similares (SESPAS, etc.)
**Evaluación**
Recuperar el estudio del grado de implantación del PAPPS en los centros de salud Medir actividades comunitarias Establecer relaciones o convenios con la administración de las CC. AA. para seguimiento y mejora de la implantación de las actividades preventivas Revisar la evidencia y actualizar conocimientos de manera continua en un sentido desmedicalizador Valorar la realización de nuevas evaluaciones de la implantación de las recomendaciones (el único programa en el mundo que lo hacía sistemáticamente) Aplicación bottom-up (de abajo arriba) que permita recoger ideas de los centros de salud sobre las actividades preventivas Unificar, facilitar registros e incluir recordatorios de las actividades preventivas con recomendación fuerte a favor en la HCAP Evaluar las actividades preventivas de forma descentralizada (en cada Comunidad Autónoma) Analizar la influencia del PAPPS en resultados en Salud
**Difusión**
Difusión por las nuevas tecnologías para llegar a los ciudadanos en actividades de prevención. Crear una o varias apps útiles para la población (versión web y dispositivo móvil) Revisión, actualización y difusión a cualquier profesional de Atención Primaria Incluir actividades preventivas terciarias y cuaternarias que den respuesta al envejecimiento de la población Mejorar su difusión en las actividades que hay que realizar y en su evaluación de los resultados Un buen plan de comunicación del plan estratégico Reforzar la marca y sus fundamentos, reforzar su visibilidad, financiar la innovación y la exploración en terrenos fronterizos Fortalecer redes autonómicas Noticias en prensa en todas las oportunidades posibles En el texto, en el contenido de las propias actualizaciones, incidir más en la presencia de algoritmos y mapas conceptuales, ágiles y atractivos para los profesionales, de capítulos fundamentales Grupos por provincias, áreas de salud... que se responsabilizaran de su defensa, difusión e implantación Crear tablas-resumen de recomendaciones por edad y sexo que puedan incorporarse a los documentos de ayuda de los ordenadores de la consulta, así como los test, recomendaciones al paciente, etc., necesarios para desarrollar la actividad
**Investigación**
Proyectos de investigación en promoción de la salud y prevención Elaborar Guías de práctica Clínica Estudiar qué implicaciones va a tener la medicina P4 (predictiva, participativa, personalizada y preventiva)
**Abogacía por la salud**
Influir: incorporar el PAPPS en la agenda política Orientación a la abogacía de la salud mediante el contacto periódico con grupos políticos en el Congreso de los diputados o en las CC. AA. para proposición de medidas legislativas de salud pública en coordinación con otras Sociedades como SESPAS, SEE, Nutrición, Socidrogalcohol, etc. Intervención en medios de comunicación para aumentar su visibilidad y el impacto poblacional de la marca PAPPS Acuerdos con el Ministerio de Sanidad y colaboración con WONCA (al menos región Europa y Sudamérica-antigua CIMF) y con la Asociación de Enfermería Comunitaria Unificar recomendaciones con otros grupos de trabajo de semFYC Unificar grados de recomendación entre los diferentes grupos de trabajo. ¿Volver a realizar auditorías de ámbito nacional sobre cumplimiento PAPPS? Programas y actividades dirigidos a actuaciones comunitarias: sacar a muchos pacientes del centro de salud

Respecto a las acciones de mejora a llevar a cabo en el PAPPS, recogidas a través de la encuesta online, respondieron 42 personas. Los resultados se exponen en la tabla 3.

**Tabla 3:** Acciones de mejora propuestas para el PAPPS

**Formación y Docencia**
Formación en metodologías evaluativas a los socios de semFYC interesados Cursos formativos sobre sistematización de las revisiones para todos los grupos Elaborar listados con “Recomendaciones No hacer” desde el punto de vista de la prevención Nuevos cursos formativos de actualización. Cursos para ligarlo con la educación poblacional (mejoraría el uso de la AP y gestión de consulta) Materiales divulgativos para profesionales y población APPs y formación on-line Talleres sobre actividades preventivas Cursos on-line de PAPPS para dar a conocer a residentes y Jóvenes Médicos de Familia Docencia en pregrado y en unidades docentes. Darse a conocer también a los estudiantes de Medicina. Lanzar “píldoras periódicas” con recomendaciones concretas Mesa en el Congreso de la semFYC Blog de Salud y Prevención PAPPS Vídeos educativos a profesionales y pacientes
**Coordinación**
Mayor coordinación e interacción entre grupos con competencias comunes Reunión anual de todos los grupos conjuntamente Reafirmar más los grupos de trabajo Coordinación con otras sociedades científicas para conseguir amplios consensos sobre temas controvertidos Promover la participación de asociaciones de pacientes Compartir el PAPPS con sociedades de enfermería Establecer relaciones más estrechas con las organizaciones que tengan objetivos similares (SESPAS, etc.)
**Evaluación**
Recuperar el estudio del grado de implantación del PAPPS en los centros de salud Medir actividades comunitarias Establecer relaciones o convenios con la administración de las CC.AA. para seguimiento y mejora de la implantación de las actividades preventivas Revisar la evidencia y actualizar conocimientos de manera continua en un sentido desmedicalizador Valorar la realización de nuevas evaluaciones de la implantación de las recomendaciones (el único programa en el mundo que lo hacía sistemáticamente) Aplicación bottom-up (de abajo arriba) que permita recoger ideas de los centros de salud sobre las actividades preventivas Unificar, facilitar registros e incluir recordatorios de las actividades preventivas con recomendación fuerte a favor en la HCAP Evaluar las actividades preventivas de forma descentralizada (en cada Comunidad Autónoma) Analizar la influencia del PAPPS en resultados en Salud
**Difusión**
Difusión por las nuevas tecnologías para llegar a los ciudadanos en actividades de prevención. Crear una o varias apps útiles para la población (versión web y dispositivo móvil) Revisión, actualización y difusión a cualquier profesional de Atención Primaria Incluir actividades preventivas terciarias y cuaternarias que den respuesta al envejecimiento de la población Mejorar su difusión en las actividades que hay que realizar y en su evaluación de los resultados Un buen plan de comunicación del plan estratégico Reforzar la marca y sus fundamentos, reforzar su visibilidad, financiar la innovación y la exploración en terrenos fronterizos Fortalecer redes autonómicas Noticias en prensa en todas las oportunidades posibles En el texto, en el contenido de las propias actualizaciones, incidir más en la presencia de algoritmos y mapas conceptuales, ágiles y atractivos para los profesionales, de capítulos fundamentales Grupos por provincias, áreas de salud... que se responsabilizaran de su defensa, difusión e implantación Crear tablas-resumen de recomendaciones por edad y sexo que puedan incorporarse a los documentos de ayuda de los ordenadores de la consulta, así como los test, recomendaciones al paciente, etc, necesarios para desarrollar la actividad
**Investigación**
Proyectos de investigación en promoción de la salud y prevención Elaborar Guías de práctica Clínica Estudiar qué implicaciones va a tener la medicina P4 (predictiva, participativa, personalizada y preventiva)
**Abogacía por la salud**
Influir: incorporar el PAPPS en la agenda política Orientación a la abogacía de la salud median te el contacto periódico con Grupos Políticos en el Congreso de los diputados o en las CCAA para proposición de medidas legislativas de salud pública en coordinación con otras Sociedades como SESPAS, SEE, Nutrición, Socidrogalcohol, etc Intervención en medios de comunicación para aumentar su visibilidad y el impacto poblacional de la marca PAPPS Acuerdos con el Ministerio de Sanidad y colaboración con WONCA (al menos región Europa y Sudamérica-antigua CIMF) y con la Asociación de Enfermería Comunitaria Unificar recomendaciones con otros grupos de trabajo de semFYC Unificar grados de recomendación entre los diferentes grupos de trabajo. ¿Volver a realizar auditorías de ámbito nacional sobre cumplimiento PAPPS? Programas y actividades dirigidos a actuaciones comunitarias: sacar a muchos pacientes del centro de salud

En la tabla 4 se muestran aquellas acciones o aspectos que los participantes priorizaron al considerarlas como más importantes de implementar (al menos 80% de participantes votaron dichas actuaciones).

**Tabla 4:** Orden de priorización de las acciones de mejora propuestas según fueron señaladas como prioritarias o importantes por al menos el 80% de los participantes

1. Unificar recomendaciones con otros grupos de trabajo de semFYC (PAPPS es semFYC) (97,6%)
2. Elaborar listados con «Recomendaciones No hacer» desde el punto de vista de la prevención (90,9%)
3. Influir: incorporar el PAPPS en la agenda política (90%)
4. Mayor coordinación e interacción entre grupos con competencias comunes (90%)
5. Docencia en pregrado y en unidades docentes. Darse a conocer también a los estudiantes de Medicina (88,1%)
6. Revisión, actualización y difusión a cualquier profesional de Atención Primaria (88,1%)
7. Establecer relaciones o convenios con la administración de las CC. AA. para seguimiento y mejora de la implantación de las actividades preventivas (88,1%)
8. Revisar la evidencia y actualizar conocimientos de manera continua en un sentido desmedicalizador (88,0%)
9. Valorar la realización de nuevas evaluaciones de la implantación de las recomendaciones (el único programa en el mundo que lo hacía sistemáticamente) (88,0%)
10. Cursos formativos sobre sistematización de las revisiones para todos los grupos (86,7%)
11. Crear un Blog sobre Salud y Prevención PAPPS (85,7%)
12. Analizar la influencia del PAPPS en resultados en Salud (85,7%)
13. Elaborar materiales divulgativos para profesionales y población (83,3%)
14. Realizar cursos online del PAPPS para dar a conocer a residentes y jóvenes Médicos de Familia (83,3%)
15. Crear tablas-resumen de recomendaciones por edad y sexo que puedan incorporarse a los documentos de ayuda de los ordenadores de la consulta, así como los test, recomendaciones al paciente, etc. necesarios para desarrollar la actividad (82,9%)
16. Unificar grados de recomendación entre los diferentes grupos de trabajo (82,9%)
17. En el texto, en el contenido de las propias actualizaciones, incidir más en la presencia de algoritmos y mapas conceptuales, ágiles y atractivos para los profesionales, de capítulos fundamentales (81%)
18. Formación en metodologías evaluativas a los socios de semFYC interesados (80,4%)

A modo de conclusiones, se exponen a continuación las principales estrategias que se proponen para mejorar la situación del programa en los próximos años (Matriz de estrategias):

*A.* *Estrategias defensivas (potenciar fortalezas), usar fortalezas para evitar amenazas:*
1. Generar recomendaciones del PAPPS («conocimiento PAPPS»), de forma actualizada, transparente, constante, sintética, realista y ágil, dado que es elaborado por un programa referente a nivel nacional con prestigio, dedicación e independiente sin conflictos de intereses.
2. Generar formación y difusión de estas recomendaciones y no perder el trabajo conseguido, con empoderamiento del médico de familia, utilizando su prestigio, experiencia y credibilidad y así conseguir autofinanciación.
3. Generar la participación multiprofesional, enfermeras, médicos residentes, médicos de otras especialidades, unificando recomendaciones y aumentando su difusión y aceptación.
4. Generar líneas de investigación y de evaluación que mantengan su nivel científico técnico y de calidad y mantener su prestigio, conocimiento y expansión.

*B.* *Estrategia de reorientación, ajustes internos (vencer debilidades aprovechando las oportunidades):*
1. Expansión/difusión a otros profesionales, a la administración y a la población general.
- Mejorar la difusión a otros profesionales, creando alianzas con otros grupos.
- Mejorar la difusión a la administración y a la población general.
- Adaptarse a los nuevos tiempos redes sociales (TIC) y APP.
- Nuevo formato de la página web con buscador ágil, y para formación.
- Adaptación a la historia clínica de los distintos servicios de salud de las distintas comunidades autónomas.
2. Consolidarse como referente, en promoción y prevención.
- Darse a conocer, mayor visibilidad.
- Lograr que las recomendaciones de los diversos grupos se oficialicen en el ámbito del SNS y lo asuman como propio.
- Alianzas/convergencia con otros grupos de trabajo.
- Realizar actividades dirigidas a profesionales y a la población.
3. Internacionalización, universalización del programa.
- Unificar programas preventivos en nuestro país y con otros países de nuestro entorno. Alianza con grupos afines del ámbito iberoamericano.

*C.* *Estrategia de supervivencia (evitación y rediseño), reducir al mínimo las debilidades y evitar amenazas:*
1. Promover la elaboración de recomendaciones en colaboración con otras sociedades científicas, asociaciones de pacientes, gestores y políticos.
2. Impulsar la difusión de recomendaciones aprovechando todos los canales disponibles, incluyendo redes sociales y página web, tanto de semFYC como del resto de entidades colaboradoras en la elaboración de recomendaciones.
3. Adaptar recomendaciones y difusión de las mismas a las distintas «poblaciones dianas»: MAP, FEA, MIR, DUE, población general, gestores.
4. Incorporar en cada grupo de trabajo al menos un profesional < 40 años: valorar que sea responsable de adaptación/difusión de recomendaciones en redes sociales.
5. Promover estudios de investigación para evaluar la eficiencia (en términos de resultados en salud y económicos) de las recomendaciones (incluidas las referidas a «No hacer»): dedicar el tiempo a lo «útil».

*D.* *Estrategias de potenciación (crecimiento e innovación), utilizar las fortalezas para aprovechar las oportunidades:*
5. Potenciar la visibilidad y difusión del PAPPS a nivel internacional y promover la colaboración con grupos de prevención y organismos científicos europeos y mundiales (WONCA, EUROPREV, Confederación Iberoamericana de Medicina Familiar).
6. Fomentar y expandir el conocimiento y rigor científico del PAPPS a través de actividades dirigidas a la formación de profesionales sanitarios y recomendaciones preventivas dirigidas a pacientes.
7. Transmitir la importancia de la Promoción de la Salud y la Educación para la Salud haciendo uso de la tecnología de la información y comunicación (TIC) y las redes sociales.
8. Promover el compromiso, experiencia y profesionalidad del PAPPS a través de proyectos de investigación de excelencia.

**Lo conocido sobre el tema**

- El Programa de Actividades Preventivas y de Promoción de la Salud (PAPPS) ha contribuido de manera sustancial a mejorar la calidad asistencial y el desarrollo de la Atención Primaria en España.
- Entre las recomendaciones encaminadas a promover el desarrollo del PAPPS destacan la integración del programa en el catálogo de prestaciones de los servicios de salud de las Comunidades Autónomas, el fomento en el ciudadano de los cuidados de su propia salud (*patient empowerment*), o la puesta en marcha de investigaciones con diseños más potentes para evaluar los resultados en salud de las distintas recomendaciones.
- La elevada presión asistencial, la escasez de tiempo en la consulta para administrar las recomendaciones preventivas y la excesiva burocratización han influido desfavorablemente en la transmisión de las recomendaciones preventivas en Atención Primaria.

**Qué aporta este estudio**

**-** La unificación de las recomendaciones del PAPPS con otros grupos de trabajo de semFYC y la elaboración de listados con «Recomendaciones No hacer» constituyen propuestas de mejora prioritarias del programa.
- A partir de la matriz DAFO se identifican las siguientes estrategias de mejora: 1) generar formación y recomendaciones del PAPPS de forma actualizada, fomentando la participación multiprofesional en el programa (estrategias defensivas); 2) consolidar el programa como referente en promoción y prevención, y potenciar su internacionalización (estrategias de reorientación); 3) impulsar la difusión de las recomendaciones a través de redes sociales, página web, y semFYC, y fomentar la colaboración con otras sociedades (estrategias de supervivencia); 4) transmitir la importancia de la Promoción de la Salud y la Educación para la Salud y promover el compromiso y experiencia del PAPPS a través de proyectos de investigación de excelencia (estrategias de potenciación).

## Conflicto de intereses

Los autores declaran no tener ningún conflicto de intereses.

## Figures and Tables

**Table:** 

Alejandro Villarín Castro Pablo Aldaz Herce José Luis Quintana Gómez Carlos Brotons Cuixart Patricia Alfonso Landa Elena Melús Palazón Manel Ferrán Mercadé Francisco Martín Ruiz Lavela Amando Martín Zurro Luis Ángel Pérula de Torres Ramón Ciurana Misol Carmen Moliner Prada Jesús Sueiro Justel Azucena Carrera Camarón Tomás Gómez Gascón Rodrigo Córdoba García Antonio Alberto León Martín José Javier Gómez Marco Alberto López García-Franco Miguel Ángel Acosta Benito Carmen Vela Vallespín José Ramón Vázquez Díaz Elena Muñoz Seco Begoña Bellas María del Carmen Fernández Alonso José Miguel Bueno Ortiz Ana Pilar Javierre Miranda Yolanda Herreros María Martín-Rabadán Muro José Mengual Gil Cinta Estrada Alifonso Celia Jiménez García Elena Valdivia Castro Cruz Bartolomé Moreno Nicomedes Rodríguez Rodríguez Fernando Isidro Lago Deibe Asunción Luque	Elvira Callejo Jiménez Joaquín San José Arango Jorge Navarro Pérez Enrique Martín Rioboó Maite Corredor Ibáñez Jesús López-Torres Hidalgo Pablo Alonso Coello María José Iglesias Piñeiro Juanjo Mascort Roca José Ignacio Ramírez Manent Agustina Moraleda Borlado Cristina Velarde Mayol Saturna Tierno Susana Aldecoa Landesa Francisco Martín Fernández Ana Clavería María Medina Sampedro Marina Pinilla Lafuente Antoni Sisó Almirall José Otal Bareche Vitorio Tapiz Manuel García Encabo Emilio Salguero Chaves Javier Ruiz Moruno María Pilar Arroyo Aniés Esperanza Romero Rodríguez Alicia Monzón Guerra María Reyes González Zapico Juan Quintana Pilar Buil Cosiales David Guardeño Ropero María Agrela Cardona Consuelo Corrionero Pilar Kloppe Villegas Zeneida Herrera Valladolid María Rosa Boluda Albiñana
